# EBV‐positive, HHV8‐negative large B‐cell lymphoma with an unusual germinotropic growth pattern in an immunocompetent patient

**DOI:** 10.1002/ccr3.1713

**Published:** 2018-07-29

**Authors:** Saja K. Asakrah, Parul Bhargava, Christine R. Bryke

**Affiliations:** ^1^ Department of Pathology Beth Israel Deaconess Medical Center Boston Massachusetts; ^2^ Department of Laboratory Medicine University of California San Francisco California; ^3^ Department of Cytogenetics Beth Israel Deaconess Medical Center Boston Massachusetts

**Keywords:** Epstein‐Barr virus, germinotropic, HHV8, immunocompetent, karyotype, lymphoma, nongerminal center

## Abstract

Clinicopathologic and cytogenetic findings of an unusual EBV+ve, HHV8−ve germinotropic lymphoma, with a nongerminal center immunophenotype occurring in an immunocompetent individual, are presented. A comprehensive literature search revealed a single report of three similar cases. These may represent a unique subset of EBV‐positive large B‐cell lymphomas in immunocompetent individuals.

## 1 INTRODUCTION

Several Epstein‐Barr virus (EBV)‐associated non‐Hodgkin B‐cell lymphomas have been described in the 2008 World Health Organization (WHO)^1^ classification of lymphoid disorders, and some have been further refined in the 2017 WHO update.^2^ These include entities that are EBV‐positive by definition, for example, EBV‐positive diffuse large B‐cell lymphoma, EBV‐positive mucocutaneous ulcer (provisional entity), lymphomatoid granulomatosis, and others where a significant proportion of cases have EBV expression such as Burkitt lymphoma, plasmablastic lymphoma, primary effusion lymphoma, and diffuse large B‐cell lymphoma associated with chronic inflammation. A majority of these occur in the context of primary or secondary immunodeficiency disorders and present with distinct clinicopathologic features.

Of note, EBV‐positive diffuse large B‐cell lymphoma (NOS), previously known as EBV‐positive DLBCL of the elderly, can occur in young and immunocompetent patients with no history of any immunosuppressive disorders.^3^, ^4^, ^5^ Extranodal involvement and poor clinical outcome are common. Histologically, the lymphoma can present as either a monomorphic infiltrate of large atypical cells or a polymorphic infiltrate composed of atypical cell within a reactive background. The atypical cells are, by definition, EBV‐positive. They more frequently have a nongerminal center (GC) immunophenotypic profile, lacking CD10, and expressing IRF4/MUM1. CD30 expression has been reported and is associated with a worse outcome.^6^

EBV‐positive large B‐cell lymphoma with an exclusive intrafollicular growth pattern has not been described in any of the above‐mentioned WHO entities. This pattern of growth is classically seen in HHV8‐positive, EBV‐positive, B‐cell lymphoma known as germinotropic lymphoproliferative disorder (GLPD). This lymphoproliferative disorder is seen in HIV‐negative individuals, usually presents as a lower stage disease and has an indolent clinical course. The large atypical infiltrating germinal centers have plasmablastic features, are negative for CD20, CD10, BCL6, CD138, show light chain restriction, but are polyclonal.^7^, ^8^

Although our case has a germinotropic growth pattern and expresses EBV, unlike GLPD, the tumor cells lack HHV8 and have retained their B‐cell signature, expressing B‐cell markers‐like CD20. The lymphoma cells show a non‐GC immunophenotype and high‐proliferation index. Cases with similar features have been reported in a recent study,^9^ and we believe these represent a unique entity in the spectrum of EBV‐positive B‐cell lymphoma in immunocompetent individuals.

## 2 CASE REPORT

The patient is an 84‐year‐old woman with a long‐standing history of mild leukopenia attributed to possible myelodysplastic syndrome, who presented with marked right leg swelling and hypercalcemia. Positron emission tomography (PET) scan showed FDG‐avid extensive axillary, left hilar, pelvic and inguinal lymphadenopathy. She was admitted and a lymph node biopsy from the left inguinal region was performed.

## 3 MATERIALS AND METHODS

The lymph node biopsy was received fresh in pathology and processed according to the standard lymphoma protocol at our institution. A representative portion was submitted in RPMI media and sent for flow cytometry, and a separate fresh portion sent for cytogenetic analysis. The remainder was fixed in buffered formalin and embedded in paraffin. Hematoxylin and eosin stains were performed on 5‐Mm tissue sections.

### 3.1 Immunohistochemical staining

Paraffin immunoperoxidase stains were performed on a DAKO autostainer (DAKO Agilent technologies, Santa Clara, CA, USA). The antibodies used in this study are the following: CD20, PAX5, CD30, CD10, BCL6, MUM1, CD21, CD23, CD2, CD7, CD4, CD8, MIB1 (ready to use antibodies, DAKO), CD15 (1/25 dilution, DAKO), CD3 (1/200 dilution, DAKO), CD5 (1/40 dilution, Nonvocastra Leica Biosystems Inc. 1700 Leider LaneBuffalo Grove, IL 60089 United States), HHV8 (ready to use antibody, Cell Marque, Rocklin, CA, USA), CXCL13, and PD1 performed at Neogenomics Laboratories (Fort Myers, FL, USA). In situ hybridization was performed using the DAKO probes against Epstein‐Barr virus (EBV) RNA.

### 3.2 Flow cytometry

Ten‐color flow cytometric analysis was performed on a Navios© flow cytometer (Beckman Coulter, Indianapolis, IN, USA). The following antibodies were tested: Kappa, Lambda, ckappa, clambda, and CD antigens 2, 3, 4, 5, 7, 8, 10, 11c, 19, 20, 23, 34, 38, 45, and 56. The technical processing was performed at Neogenomics Laboratories (Fort Myers, FL, USA), where the test was developed and its performance characteristics determined. The professional interpretation of this test was completed at Beth Israel Deaconess Medical Center, Boston, MA, USA.

### 3.3 Cytogenetic studies

Unstimulated and 3‐day DSP30/IL2‐stimulated (B‐cell mitogen) cultures were set up for Giemsa‐banded metaphase chromosome analysis. A total of 20 mitotic cells were examined in detail. Fluorescence in situ hybridization was performed on uncultured cells, and a total of 200 nuclei were scored for each probe set used. These included dual color break apart probe sets for BCL6 and MYC, an IGH‐BCL2 dual color dual fusion translocation probe set, and a ATM and TP53 dual color probe set from Abbott Molecular (Abbott Park, IL, USA) and a IRF4 (MUM1) dual color break apart probe set and an IGH‐CCND3 dual color dual fusion translocation probe set from Leica Biosystems (Nussloch, Germany).

## 4 RESULTS

### 4.1 Pathologic findings

**Figure 1:**
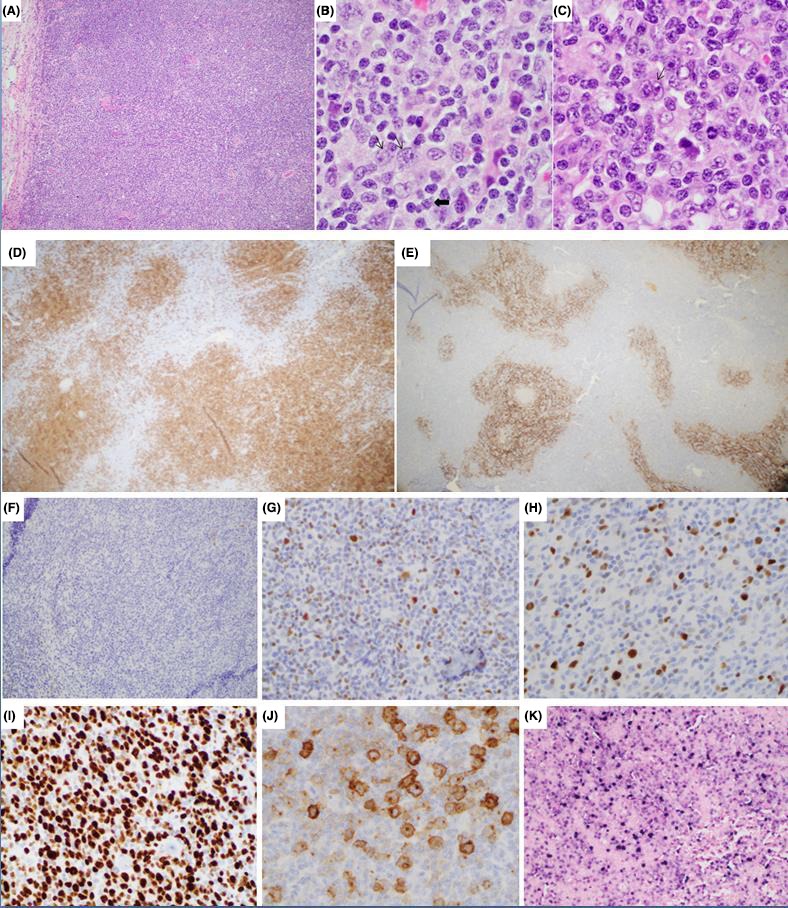
EBV‐positive large B‐cell lymphoma with intrafollicular growth pattern. Hematoxylin and eosin stains show complete effacement of the lymph node architecture by large lymphoma cells, with a vague nodular growth pattern, ×20 (A) composed of atypical centroblasts (thin arrows), centrocytes (thick arrow), ×100 (B), and Hodgkin‐like cells (thin arrow) (C). By immunohistochemical stains, pan B‐cell marker CD20 highlights the nodular arrangement of neoplastic B cells (D) and CD23 (E) highlights prominent follicular dendritic meshwork throughout, ×20. The neoplastic cells are negative for CD10, ×20 (F), partially positive BCL6, ×50 (G), and MUM1, ×50 (H). Ki‐67 proliferation index is high, ×50 (I). A subset of the atypical B‐cells express CD30 in a Golgi‐like pattern, ×100 (J). In situ hybridization for Epstein‐Barr virus encoded RNA (EBER) shows positivity in large lymphoma cells, ×20 (K)

The lymph node (see Figure 1) architecture was effaced by vaguely nodular large cell aggregates composed mainly of large atypical centroblast‐like cells. Occasional polylobulated cells and cells with prominent cherry‐red nucleoli (Reed‐Sternberg‐like cells) were seen. Variably abundant background small lymphocytes with irregular nuclear outlines were present. Occasional mitoses and apoptotic cells were seen.

By immunohistochemical staining, the clusters of large cells were diffusely immunoreactive for pan B‐cell markers (CD20, PAX5). These B cells coexpress CD30, but were nonimmunoreactive for CD15 and ALK. They were CD10(−), with a subset expressing BCL6 and MUM1, in keeping with a nongerminal center immunophenotype (by Hans classifiers). The B‐cell‐rich nodular areas had background intact CD21 and CD23 immunoreactive follicular dendritic meshwork as well as admixed background small lymphocytes that were CXCL13 and PD1‐positive, in keeping with overrun germinal centers. The atypical large B cells were HHV8‐negative, but expressed EBV‐encoded RNA (ie, EBER+). No immunoreactivity was seen by in situ hybridization studies for kappa or lambda. A majority of the background small lymphocytes were T cells (CD2+, CD3+, CD5+, CD7+), predominantly CD4+, with fewer CD8+ T cells. By Ki‐67 staining, the proliferation index within the large B cells was approximately 80%.

### 4.2 Flow cytometry

The concurrent flow cytometry did not detect clonal B cells by either light chain surface expression or cytoplasmic expression.

### 4.3 Cytogenetics

**Figure 2:**
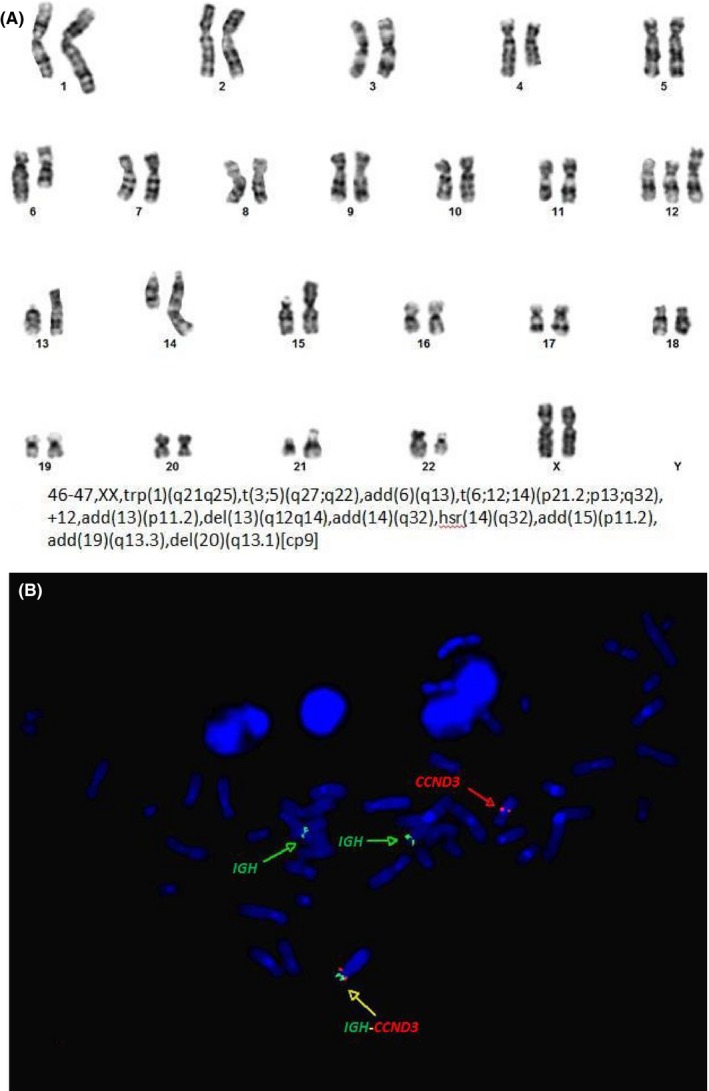
Cytogenetic findings of EBV‐positive large B‐cell lymphoma with intrafollicular growth pattern. A, Representative abnormal metaphase cell with 6q−, trisomy 12, 13q−, a variant of the t(6;14)(p21;q32) translocation also involving chromosome 12, and other chromosome aberrations. B, FISH with an *IGH‐CCND3* dual color dual fusion probe set (Leica Biosystems) performed on the same metaphase cell. An *IGH‐CCND3* fusion signal due to a t (6;12;14)(p21;p13;q32) is present on the short arm of the derivative chromosome 6 (yellow arrow). A normal red *CCND3* signal is on the chromosome 6 with a deletion of the long arm. Green *IGH* signals are on the derivative chromosome 14 and the normal chromosome 14

Nine of the 20 metaphase lymph node cells analyzed had complex abnormal karyotypes. A composite karyotype of the cells is: 46‐47,XX,trp(1)(q21q25),t(3;5)(q27;q22),add(6)(q13),t(6;12;14) (p21.2;p13;q32),+12,add(13)(p11.2),del(13)(q12q14),add(14)(q32),hsr(14)(q32),add(15)(p11.2), add(19)(q13.3),del(20)(q13.1)[cp9]. Among the many aberrations were 6q−, trisomy 12, 13q−, and a variant of the t (6;14)(p21;q32) translocation also involving chromosome 12 that FISH demonstrated resulted in the IGH‐CCND3 gene rearrangement. Figure 2 shows a representative G‐banded metaphase cell and FISH with an IGH‐CCND3 dual color dual fusion probe set (Leica Biosystems) performed on the same cell. An IGH‐CCND3 fusion signal due to a t (6;12;14)(p21;p13;q32) is present on the short arm of the derivative chromosome 6.

Approximately 15% of the uncultured interphase lymph node cells examined by FISH had an extra intact BCL6 signal, trisomy 12, deletion 13q14, and an IGH‐CCND3 fusion signal. There was no evidence of the IGH‐BCL2 gene rearrangement or rearrangements of the BCL6, MYC, and IRF4 (MUM1) genes. There was also no evidence of deletion of the ATM and TP53 genes.

## 5 CLINICAL FOLLOW‐UP

The patient was treated with rituximab initially while awaiting final pathology report. Once lymphoma was confirmed, she received six cycles of rituximab combined with low‐dose of doxorubicin, cyclophosphamide, vincristine, and prednisone (R‐mini‐CHOP). Her chemotherapy course was complicated by pneumonia and respiratory failure after cycle 1. This was managed by antibiotics and resulted in a delay in cycle 2. She also had a delay in cycle 5 due to influenza B requiring admission.

Positron emission tomography scans following cycle 6 showed significantly improved pelvic adenopathy, and resolution of splenomegaly. However, increased uptake in the right axillary nodes was noted, for which she received local radiotherapy. The patient was last seen at a follow‐up visit 1 month after the completion of radiation treatment. She was stable and in clinical remission.

## 6 DISCUSSION

We describe a rare presentation of an EBV (+) HHV8(−)germinotropic large B‐cell lymphoma. A comprehensive work‐up including immunohistochemistry, flow cytometry, and cytogenetic analysis was performed. Our patient is HIV(−) with a clinical suspicion of low‐grade myelodysplasia and no other comorbidities. The lymphoma has a nongerminal center immunophenotype (by Hans classifiers). Cytogenetic analysis showed a complex abnormal karyotype including a variant of the t(6;14)(p21.2;q32) translocation which resulted in an IGH‐CCND3 gene rearrangement.

We conducted a comprehensive literature search to look for similar cases. Mackrides et al^10^ tested EBV expression in 382 cases of follicular lymphoma and identified 10 EBV‐positive follicular lymphomas (prevalence 2.6%). About 80% of these patients were immunocompetent and presented with lymphadenopathy and initial clinical stage between IIA and IVA. A diagnosis of follicular lymphoma was rendered based on the immunophenotype and follicular distribution. All the EBV‐positive cases were CD10‐positive and coexpressed BCL2. Most of these cases also expressed BCL6. CD30 expression was found in four of the 10 cases and demonstrated a strong intrafollicular expression with a distribution similar to the EBER expression. No Hodgkin‐Reed‐Sternberg‐like cells were reported. FISH studies for t(14;18) were available for only two cases which was positive in one case and negative in the other. Interestingly, 70% (7/10) of the EBV‐positive follicular lymphoma in this study showed high‐grade features (3A/3B) and the remainder 30% initially composed low‐grade histological criteria (grade 1‐2) then progressed to either high‐grade follicular lymphoma (3A) or diffuse large B‐cell lymphoma. Our case shares some histological and immunophenotypic features with some of the cases reported in the above study, including restricted intrafollicular growth pattern with increased atypical centroblasts, EBER, and CD30 expression. However, unlike the above cases, the morphologic findings are not that of a follicular lymphoma. The malignant intrafollicular B cells in our case are CD10 negative and have a nongerminal center immunophenotype with partial expression of MUM1 and BCL6. No BCL2 rearrangement was seen on cytogenetic analysis. Therefore, a diagnosis of EBV‐positive follicular lymphoma is unlikely in our case.

In another recent study, Lorenzi et al^9^ reported three cases of EBV‐positive intrafollicular lymphoma with nongerminal center immunophenotype and considered them as a variant of germinotropic lymphoproliferative disorder. Similar to our case, all three patients reported by Lorenzi were immunocompetent and presented with an advanced clinical stage (IIIsB‐IVB). The lymph node architecture in all three cases was completely or partially effaced by a nodular lymphoid infiltrate. No normal follicles were identified. The atypical follicles were composed of large cells including immunoblasts, centroblasts, and Hodgkin‐Reed‐Sternberg‐like cells. All follicles had follicular dendritic meshwork that variably expressed CD21, CD23, CXCL13, Clusterin, Claudin 4, and EGFR. The atypical cells were strongly positive for B‐cell marker CD20 with coexpression of CD30, MUM1, and CXCR5. CD10 was mostly negative and showed <25% positivity in one of the cases. BCL6 staining was variable among the cases. EBV was expressed mainly within the atypical follicles. Ki‐67 proliferation index was high within the follicles and approached 70%. FISH analysis reported for *BCL2, MYC, BCL6,* and *PAX5* genes did not show any chromosomal rearrangements or other aberration. By immunohistochemistry, atypical large B cells showed increased c‐MYC and STAT3 expression. Similar to the cases reported by Lorenzi et al, the B‐cell lymphoma in our case shows a germinotropic growth pattern, has a nongerminal center immunophenotype by Hans algorithm and a high‐proliferation index by Ki‐67 (80%). We have summarized the clinicopathologic features of all these four reported cases in Table 1. The present case additionally had fresh tissue submitted for metaphase chromosome analysis which showed a complex abnormal karyotype with a rearrangement of chromosome 3 resulting in gain of BCL6, trisomy 12, deletion 13q, a translocation involving chromosomes 6, 12, and 14 resulting in the IGH‐CCND3 gene rearrangement, and several other chromosome aberrations (see Figure 2).

**Table 1:** Summary of clinicopathologic findings of reported patients with germinotropic, EBV (+), HHV8 (−) lymphomas

Reference	Age/Sex	Clinical	EBV	HHV8	B‐cell markers	Immunoglobulin light chains	COO	HIV	Treatment	Follow‐up
Virchows Arch (2016) 468:441‐450	66/M	Mediastinal, retroperitoneal, axillary, inguinal LAD, splenomegaly	+	−	CD20, PAX5,	Polyclonal	Non‐GC	−	R‐CHOP	DOD, 18 m
Virchows Arch (2016) 468:441‐450	77/F	Cervical & abdominal LAD, splenomegaly, bone	+	−	CD20, PAX5,	Kappa restricted	Non‐GC	−	R	AWD, 12 m
Virchows Arch (2016) 468:441‐450	63/F	Supra‐diaphragmatic & mesenteric LAD, bone	+	−	CD20, PAX5,	Polyclonal	Non‐GC	−	R‐CHOP	NED, 28 m
Our case	84/F	Axillary, left hilar, pelvic and inguinal lymphadenopathy	+	−	CD20+, PAX5+	No surface or cytoplasmic immunoglobulin	Non‐GC	−	R‐CHOP & local radiation	NED, 1 m

AWD, alive and well with disease; CHOP, cyclophosphamide, doxorubicin, vincristine, prednisone; COO, cell of origin; DOD, dead of disease; NED, no evidence of disease; GC, germinal center; HIV, human immunodeficiency virus; R, rituximab.

In conclusion, we report a rare case of a high‐grade B‐cell lymphoma with an exclusive intrafollicular growth pattern that is EBV‐positive, HHV8‐negative, in an immunocompetent patient. A comprehensive work‐up including karyotypic analysis is presented. The recent WHO 2016 update does not describe such cases and no specific categorization for such cases exists. Together with the three cases reported by Lorenzi et al, such cases may represent a unique subtype of EBV‐positive, HHV8‐negative diffuse large B‐cell lymphoma.

## CONFLICT OF INTEREST

None declared.

## AUTHORSHIP

PB (primary hematopathologist): evaluated the histopathology of the lymph node, conducted immunophenotypic work‐up, and formulated the pathologic diagnosis. She helped in writing and editing the case report. SA (pathologist): gathered all the clinicopathologic information regarding the case and took photomicrographs. She wrote the primary draft of the manuscript. CB (primary cytogeneticist): evaluated the karyotypic findings and conducted further FISH work‐up on tissue from this case. She provided the cytogenetic findings on the case including the corresponding karyotype and FISH images.
